# Supplementary material for: The Incidence and Risk Factors for Enterotoxigenic E. coli Diarrheal Disease in Children under Three Years Old in Lusaka, Zambia
Source: Microorganisms. 2024 Mar 29;12(4):698. doi: 10.3390/microorganisms12040698 (PMC11051722; doi:10.3390/microorganisms12040698)
Supplement: Supplementary file 1 [file microorganisms-12-00698-s001.zip › Supplementary File S1 .pdf]

Supplementary Data.

ETEC diarrhoea seasonality

The seasonality of ETEC infection among the children who were positive for ETEC, excluding children with repeated infections (n=121) are presented by site in Figure 3. As for diarrhoea, transmission of ETEC was observed across the year and with the highest number observed during the hot and rainy season. (i.e. October- December)

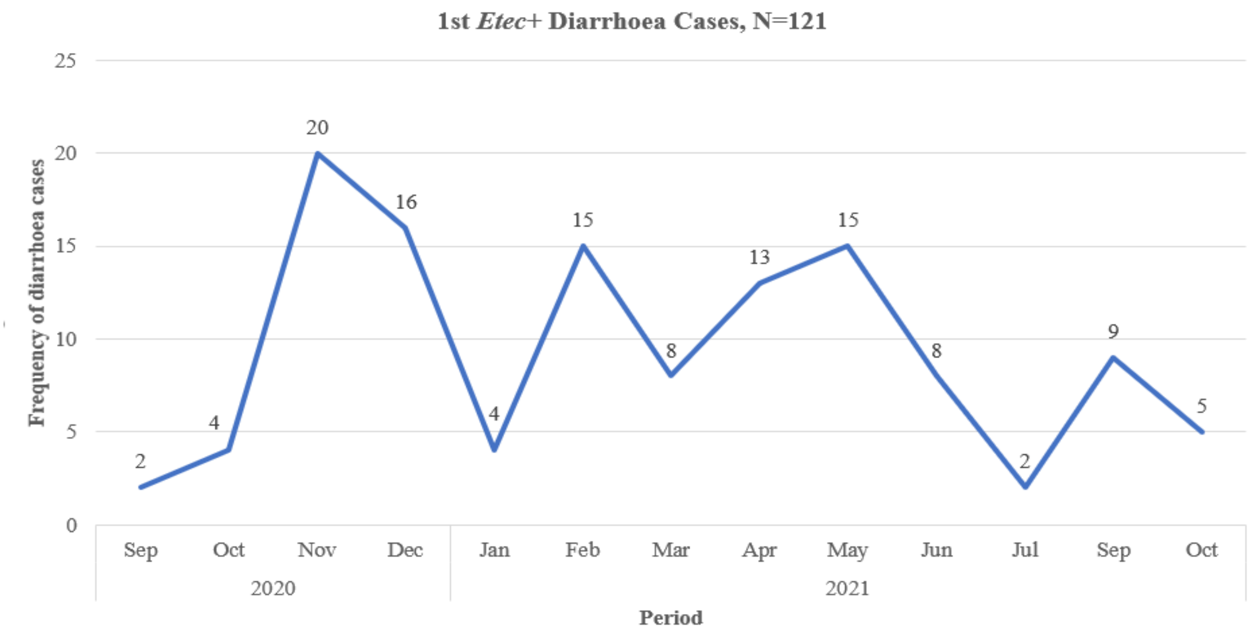

Figure S1. Number of ETEC positive diarrhoea cases (culture test result, 1st diarrhoea episode) per month, between Sept 2020 and Oct 2021
