# Supplementary material for: The Incidence and Risk Factors for Enterotoxigenic E. coli Diarrheal Disease in Children under Three Years Old in Lusaka, Zambia
Source: Microorganisms. 2024 Mar 29;12(4):698. doi: 10.3390/microorganisms12040698 (PMC11051722; doi:10.3390/microorganisms12040698)
Supplement: Supplementary file 1 [file microorganisms-12-00698-s001.zip › Supplementary File S2.pdf]

Primers

Primers	Sequence	References
LTF:	ACG GCG TTA CTA TCC TCT C	[13]
LTR:	TGG TCT CGG TCA GAT ATG TG	
SThnyF:	TTCACCTTTCCCTCAGGATG	
SThnyR:	CTATTCATGCTTTCAGGACCA	
STpF1	TCT TTC CCC TCT TTT AGT CAG	
STpR2	ACA GGC AGG ATT ACA ACA AAG	

PCR conditions

**Multiplex PCR STh STp LT Mastermix**		1 reaction
		µl
Template (DNA rapid boil bact)		*1*
Primer LT F (10mM)		*0.5*
Primer LT R (10mM)		*0.5*
Primer STp F (10mM)		*0.5*
Primer STp R (10mM)		*0.5*
Primer STh F (10mM)		*1*
Primer STh R (10mM)		*1*
MgCl2 (25mM)		*0.4*
Readymix (x2)		*10*
Water		*4.6*
Total volume		***20***
Program		
95⁰C	*2 min*	
95⁰C	*15s*	
52⁰C	*8s*	
72⁰C	*10s*	*30 cycles*
72⁰C	*2 min*	
